# Comparing Aberration Detection Methods with Simulated Data

**DOI:** 10.3201/eid1102.040587

**Published:** 2005-02

**Authors:** Lori Hutwagner, Timothy Browne, G. Matthew Seeman, Aaron T. Fleischauer

**Affiliations:** * Centers for Disease Control and Prevention, Atlanta, Georgia, USA

**Keywords:** outbreak detection, surveillance, aberration detection

## Abstract

We compared aberration detection methods requiring historical data to those that require little background by using simulated data. Methods that require less historical data are as sensitive and specific as those that require 3–5 years of data. These simulations can determine which method produces appropriate sensitivity and specificity.

The Early Aberration Reporting System (EARS) was developed to allow analysis of public health surveillance data. Several alternative aberration detection methods are available to state and local health departments for syndromic surveillance. Before 2001, most statistical aberration detection methods required at least 5 years of background data (*1**–**6*). However, with the release of *Bacillus anthracis* in the U.S. mail shortly after the September 11, 2001, World Trade Center attacks, substantial interest has emerged in public health tools that could be rapidly implemented without requiring years of background data. Newly developed nonhistorical aberration detection methods can require as little as 1 week of data to begin analysis, although they have not been extensively evaluated against traditional historical methods (*7**,**8*).

The objective of our study was to determine the sensitivity, specificity, and time to detection of 3 methods that require <3 years of historical baseline data, C1–MILD (C1), C2–MEDIUM (C2), and C3–ULTRA (C3), and compare the results with those of 2 methods that require 5 years of historical baseline, the historical limits method (*2*) and the seasonally adjusted cumulative sum (CUSUM) (*5*), based on simulated data. Simulated data were used to avoid some of the interpretation difficulties that can come from making these comparisons on the basis of empirically observed, natural disease data. All 5 of these methods are components of EARS (*7*).

## The Study

The methods C1, C2, and C3 were named according to their degree of sensitivity, with C1 being the least sensitive and C3 the most sensitive. All 3 methods are based on a positive 1-sided CUSUM calculation. For C1 and C2, the CUSUM threshold reduces to the mean plus 3 standard deviations (SD). The mean and SD for the C1 calculation are based on information from the past 7 days. The mean and SD for the C2 and C3 calculations are based on information from 7 days, ignoring the 2 most recent days. These methods take into consideration daily variation because the mean and SD used by the methods are based on a week's information. These methods also take seasonality into consideration because the mean and SD are calculated in the same season as the data value in question.

Since 1989, results from the historical limits method have been used to produce Figure 1 in the Morbidity and Mortality Weekly Report. This method compares the number of reported cases in the 4 most recent time periods for a given health outcome with historical incidence data on the same outcome from the preceding 5 years; the method is based on comparing the ratio of current reports with the historical mean and SD. The historical mean and SD are derived from 15 totals of 3 intervals (including the same 4 periods, the preceding 4 periods, and the subsequent 4 periods over the preceding 5 years of historical data).

The seasonally adjusted CUSUM method is based on the positive 1-sided CUSUM where the count of interest is compared to the 5-year mean and the 5-year SD for that period. The seasonally adjusted CUSUM was originally applied to laboratory-based *Salmonella* serotype data.

To calculate sensitivity, specificity, and time to detection, all 5 detection methods of EARS were used to independently analyze 56,000 sets of artificially generated case-count data based on 56 sets of parameters. These 56 sets of parameters each generated 1,000 iterations of 6 years of daily data, 1994–1999, by using a negative binomial distribution with superimposed outbreaks. Means and standard deviations were based on observed values from national and local public health systems and syndromic surveillance systems. Examples of the data included national and state pneumonia and influenza data and hospital influenzalike illness. Adjustments were made for days of the week, holidays, postholiday periods, seasonality, and trend. Any 6 years could be used, but the years 1994–1999 were used to set day of the week and holiday patterns and to avoid any problems that programs might have with the year 2000. Fifty (89%) of these datasets then had outbreaks superimposed throughout the data. Three types of outbreaks were used, each representing various types of naturally occurring events: log normal, a rapidly increasing outbreak; inverted log normal, a slowly starting outbreak; and a single-day spike. These types of outbreaks were combined with different SDs and incubation times to create 10 different types of outbreaks that had equal probability of being included in the simulated data. A year of final simulated data can be seen in the Figure, with original data and outbreaks that were added. As a result of these analyses, the statistically marked aberrations, or flags, produced by the 5 detection methods were evaluated for their specificity, sensitivity, and time to detection. These data can be obtained at http://www.bt.cdc.gov/surveillance/ears/datasets.asp.

**Figure:**
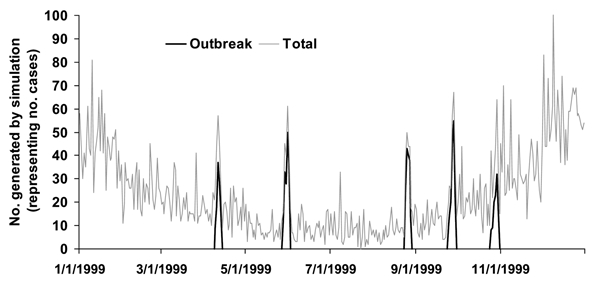
Example of 1 year of simulated data with simulated outbreaks. Simulated data are based on real means and standard deviations with different types of simulated outbreaks randomly inserted.

In our study, sensitivity was defined as the number of outbreaks in which >1 day was flagged, divided by the total number of outbreaks in the data. An outbreak was defined as a period of consecutive days in which varying numbers of aberrant cases were added to the baseline number of cases. An outbreak had days before and after it when no aberrant cases were added to the baseline case counts. Specificity was defined as the total number of days that did not contain aberrant cases (and that were not flagged), divided by the total number of days that did not contain aberrant cases. Based on these definitions, actual values for sensitivity and specificity were calculated.

Time to detection was defined as the number of complete days that occurred between the beginning of an outbreak and the first day the outbreak was flagged. For example, if a method flags an outbreak on the first day, its time to detection is 0. Likewise, if it flags on the second day, its time to detection is 1, and so on. Time to detection is an average of the times to detection for each outbreak and dataset. Only outbreaks that were flagged on at least 1 day were included in the average. Therefore, sensitivity is needed to completely interpret time to detection. We calculated 2-sided 95% confidence values, and they were relatively small and consistent.

Overall, the CUSUM methods (the seasonally adjusted CUSUM, C1, C2, and C3) had similar times to detection, but their sensitivities varied (Table). Specifically, C1, C2, and C3 showed increasing sensitivity from 60% to 71% to 82%, respectively. The seasonally adjusted CUSUM and C3 methods had similar sensitivities, 82.5% and 82.3%, but C3 had a higher specificity, 88.7% and 95.4%. The historical limits and C1 and C2 methods showed varying sensitivities (44%–71%), with C1 and C2 having the highest, but all demonstrated similar specificities (96%–97%).

When results were stratified by outbreak type, 1-day outbreaks (i.e., spikes) exhibited the lowest sensitivities. Analysis was broken down by dataset and outbreak type (Table A1 and Table A2).

For the 6 datasets that contained noise but no outbreaks, no sensitivity or time to detection exist to calculate. The overall specificity for the seasonally adjusted CUSUM, historical limits, C1, C2, and C3 were 88.7%, 98.3%, 97.2%, 97.2%, and 95.2%, respectively. The specificity for these 6 datasets was consistent with general results. The historical limits method showed superior specificity in all but the last dataset.

## Conclusions

These simulations demonstrate that the methods for aberration detection that require little baseline data, C1, C2, and C3, are as sensitive and specific as the historical limits and seasonally adjusted CUSUM methods. As expected, C1, C2, and C3 showed increasing sensitivities in accordance with their intended sensitivity levels (C1 being the least sensitive, C3 being the most), but with decreasing specificities as sensitivity increases. Seasonally adjusted CUSUM and the historical limits method also showed sensitivities and specificities as expected, with the seasonally adjusted CUSUM having the lower specificity and higher sensitivity. These findings emphasize the effectiveness of aberration detection methods without requiring long-term historical data as a baseline.

Since the 10 simulated outbreaks were randomly generated by using consistent rates, the sensitivity, specificity, and time to detection could be stratified by dataset and outbreak type. The results of these analyses were largely congruent with the expected findings, with some variations. The simulated datasets are designed for public health officials to select a dataset that best reflects their data of interest or the type of outbreak they are anticipating to determine which method provides them with the sensitivity and specificity they would find useful. The simulated datasets can also be used to make comparisons with other methods.

The aberration detection methods C1, C2, and C3 are used in several states, counties, and local public health departments. Public health departments are able to apply these methods to data sources that do not have long periods of baseline data. Public health departments are also able to apply 1 set of methods they understand to various types of diseases, covering different frequencies and seasonalities. The C1, C2, and C3 methods have detected outbreaks of public health interest, including West Nile disease and the start of the influenza season.

C1, C2, and C3 demonstrate consistency over the various situations represented in these simulations. Other aberration detection methods exist, as do other simulated datasets. The simulated datasets presented in this paper cover a larger variety of types of data that might be expected in public health. These simulated datasets also include enough past years of data so that methods that require 5 years of historical information can also be used in the comparisons. These simulations provide a method to fairly compare other methods among themselves and to the methods included in EARS.

The simulations were based on means and SDs to help determine which method performs better under which circumstances. When deciding which method to use, the potential user should base the decision on the sensitivity or specificity or the time to detection.

A potential limitation is that the method for calculating average times to detection disregards undetected outbreaks. Therefore, times to detection should not be considered without also taking into account the sensitivity. However, this method was preferred over the alternative of assigning arbitrary numbers of days to detection for outbreaks that were not detected since the alternative method could lead to misinterpretation of the data. Another limitation is that the artificial datasets may not fully reproduce the nuances of natural disease occurrences. While approximations, the simulated data were generated based on naturally observed data and included variations for trend over time, days of the week, seasons, and holidays. Therefore, while these comparisons represent relative sensitivities, specificities, and times to detection, we do not know whether results using naturally occurring data would be consistent.

The results of this study suggest that the EARS historical methods do not have a strong advantage when compared with nonhistorical methods. In fact, the lack of historical data does not impair the EARS outbreak detection methods. This study also demonstrates the effectiveness of artificial outbreak data in comparing and evaluating outbreak detection methods. As aberration detection methods are increasingly being used by state and local health departments to monitor for naturally occurring outbreaks and bioterror events, this study contributes to the quest to determine the most efficient method for analyzing surveillance data.

## Appendix

**Table A1:** Sensitivity (sen), specificity (spe) and time to detection (ttd) by method and dataset; to make further comparisons, data can be downloaded by dataset name from http://www.bt.cdc.gov/surveillance/ears/.

Dataset*	Trend†	Seas‡	Mean§	SD§	Seasonally adjusted CUSUM¶			Historical Limits#			C1–MILD**			C2–MEDIUM††			C3–ULTRA‡‡		
					sen (%)	spe (%)	ttd§§	sen (%)	spe (%)	ttd§§	sen (%)	spe (%)	ttd§§	sen (%)	spe (%)	ttd§§	sen (%)	spe (%)	ttd§§
S01	Yes	Low	90	33	96.55	67.54	0.49	79.10	90.64	2.00	42.80	97.47	1.78	54.37	97.59	1.98	71.54	96.05	1.93
S02	No	Med	30	6	70.45	98.74	2.32	7.00	99.77	5.60	50.56	98.10	1.53	64.38	98.14	1.79	79.66	96.54	1.70
S03	No	Low	1	6	94.58	86.49	0.64	69.67	96.84	2.53	72.68	90.91	0.95	79.07	91.20	0.98	83.58	90.98	0.99
S04	Yes	Very	6	4	95.49	83.82	0.68	70.75	96.41	2.41	75.36	97.16	0.92	87.31	96.94	1.06	92.53	95.04	0.97
S05	No	Very	38	13	86.98	93.95	1.42	43.00	99.79	4.05	52.38	97.50	1.51	66.23	97.46	1.73	80.21	95.64	1.62
S06	Yes	Very	1	6	92.98	90.41	0.64	69.79	98.32	2.24	81.23	94.76	0.42	88.83	94.29	0.57	93.03	92.91	0.56
S07	No	Low	150	27	57.63	98.88	2.76	1.64	100.00	6.04	39.77	98.10	1.77	53.11	98.16	2.05	71.23	96.59	1.97
S08	No	Very	90	33	88.58	95.06	1.17	51.69	99.61	3.50	63.59	97.48	1.09	78.81	97.46	1.32	87.41	95.73	1.18
S09	Yes	Low	30	6	98.70	64.49	0.29	80.00	96.89	2.03	75.36	98.38	1.04	89.48	98.42	1.21	93.45	96.94	1.02
S10	No	Low	30	6	67.30	98.32	2.42	5.34	99.68	5.65	45.42	98.00	1.67	57.74	98.05	1.89	75.28	96.51	1.85
S11	Yes	Med	301	79	99.16	22.41	0.06	94.45	46.78	0.48	62.23	98.09	1.03	79.70	98.12	1.28	86.68	96.52	1.12
S12	Yes	Med	6	4	88.12	96.69	1.40	48.91	99.64	3.81	74.44	96.01	0.97	83.74	96.00	1.09	89.75	94.56	1.02
S13	No	Very	150	27	68.41	99.17	2.33	8.97	100.00	5.53	51.64	98.09	1.40	67.87	98.09	1.66	82.41	96.37	1.59
S14	No	Med	38	13	83.39	93.32	1.66	31.30	99.89	4.59	43.41	97.54	1.80	55.11	97.64	1.99	72.35	96.03	1.93
S15	Yes	Low	6	4	99.64	27.15	0.10	96.91	35.95	0.41	47.66	97.06	1.75	57.93	97.18	1.90	73.14	95.62	1.87
S16	No	Low	6	4	92.80	83.13	0.97	62.86	96.88	2.94	50.12	95.83	1.67	57.75	95.97	1.83	71.35	94.55	1.80
S17	Yes	Very	90	33	90.31	91.59	0.77	63.72	98.86	2.66	73.89	97.52	0.72	85.20	97.37	0.89	89.09	95.43	0.79
S18	Yes	Low	75	21	83.87	92.74	1.56	30.40	99.91	4.54	41.14	97.74	1.80	52.65	97.81	2.05	70.43	96.22	2.00
S19	Yes	Very	90	33	81.62	99.62	1.59	41.78	99.86	3.94	80.28	96.85	0.61	90.33	96.57	0.76	93.14	94.81	0.66
S20	No	Med	1	6	93.70	91.14	0.72	70.80	99.01	2.29	76.67	93.83	0.48	82.81	93.62	0.63	88.78	92.87	0.68
S21	Yes	Very	75	21	91.76	92.69	1.06	54.16	99.55	3.42	62.29	97.81	1.19	78.50	97.65	1.41	87.29	95.77	1.28
S22	No	Low	38	13	83.81	92.48	1.51	35.26	99.82	4.44	44.39	97.46	1.76	55.07	97.57	1.97	72.47	95.97	1.94
S23	Yes	Med	38	13	96.13	76.30	0.59	70.19	97.07	2.68	50.55	97.69	1.51	64.98	97.71	1.71	79.93	95.95	1.65
S24	No	Very	3	2	95.82	82.35	0.63	72.68	95.66	2.29	73.97	96.87	1.02	84.61	96.80	1.14	90.56	95.13	1.05
S25	No	Low	90	33	84.45	91.58	1.41	38.40	99.82	4.20	43.23	97.30	1.76	53.61	97.40	1.92	70.81	95.85	1.90
S26	Yes	Low	75	21	39.47	100.00	2.86	8.56	100.00	5.87	96.59	96.53	0.04	97.28	96.68	0.07	97.35	95.32	0.07
S27	Yes	Very	150	27	39.12	100.00	2.83	7.25	100.00	5.82	93.24	97.44	0.18	95.85	96.74	0.23	96.37	94.52	0.21
S28	No	Low	75	21	81.26	94.72	1.78	23.43	99.96	4.85	41.84	97.74	1.84	53.00	97.80	1.98	71.54	96.21	1.96
S29	No	Med	150	27	57.36	99.10	2.75	1.72	100.00	5.93	41.77	98.09	1.71	54.86	98.18	1.92	73.44	96.57	1.90
S30	No	Med	150	27	60.48	99.13	2.61	2.52	100.00	6.07	44.31	98.09	1.59	57.57	98.13	1.85	76.82	96.52	1.83
S31	Yes	Low	150	27	43.27	100.00	2.84	9.41	100.00	5.91	95.65	97.72	0.13	97.30	97.80	0.16	97.38	96.31	0.14
S32	Yes	Low	301	79	54.46	99.62	2.79	5.18	100.00	5.51	55.46	97.56	1.29	71.41	97.70	1.55	83.86	96.17	1.45
S33	Yes	Med	30	6	83.53	98.05	1.75	24.05	99.85	4.68	59.93	98.22	1.34	76.47	98.19	1.55	87.59	96.45	1.43
S34	Yes	Low	3	2	88.66	96.47	1.37	44.46	98.84	3.88	85.26	93.46	0.78	90.98	93.64	0.85	93.94	92.65	0.77
S35	No	Very	6	4	92.46	86.87	0.99	61.42	98.47	3.17	53.22	96.79	1.55	66.86	96.84	1.68	79.88	95.28	1.62
S36	Yes	Med	150	27	93.28	90.21	0.93	49.36	99.76	3.83	54.55	98.16	1.30	71.04	98.21	1.56	84.52	96.55	1.47
S37	Yes	Low	3	2	97.15	64.58	0.45	81.88	85.24	1.67	46.60	96.01	1.75	54.49	96.11	1.86	69.87	94.60	1.86
S38	Yes	Very	30	6	67.25	100.00	2.22	21.47	99.98	4.73	92.82	97.74	0.30	96.46	97.04	0.37	97.02	95.18	0.32
S39	Yes	Med	3	2	93.60	90.28	0.97	60.27	98.51	3.06	68.82	95.82	1.18	79.02	95.89	1.28	87.15	94.46	1.21
S40	No	Very	3	2	94.07	88.53	0.82	65.31	98.22	2.72	70.99	96.58	1.11	80.98	96.52	1.22	88.63	94.90	1.13
S41	No	Med	3	2	94.43	87.00	0.82	64.58	97.88	2.86	64.91	96.00	1.26	75.00	96.08	1.40	85.45	94.58	1.35
S42	No	Med	75	21	81.73	95.86	1.74	27.12	99.96	4.62	47.58	97.75	1.56	61.88	97.77	1.74	77.46	96.09	1.69
S43	No	Very	75	21	85.26	96.00	1.54	35.59	99.89	4.24	53.46	97.76	1.42	68.39	97.75	1.62	82.17	95.93	1.53
S44	No	Very	301	79	77.68	96.03	1.77	25.98	99.96	4.55	43.66	97.77	1.62	57.43	97.81	1.82	73.69	96.15	1.73
S45	No	Med	38	13	84.90	92.79	1.49	36.94	99.83	4.30	45.06	97.50	1.75	56.52	97.60	1.91	73.64	96.03	1.83
S46	No	Low	3	2	91.95	82.44	0.88	63.04	95.61	2.73	51.88	94.71	1.52	59.13	94.79	1.65	71.56	93.39	1.60
S47	Yes	Med	75	21	85.95	92.50	1.43	35.59	99.88	4.39	44.91	97.82	1.69	57.02	97.87	1.94	74.66	96.25	1.91
S48	Yes	Very	301	79	87.42	93.90	0.94	56.07	99.36	3.09	68.14	97.86	0.85	82.38	97.75	1.08	87.58	95.80	0.93
S49	Yes	Med	1	6	93.90	85.16	0.71	71.25	97.17	2.32	71.01	93.88	0.85	79.66	93.88	0.95	86.39	92.79	0.94
S50	No	Low	301	79	73.52	96.11	2.07	14.94	99.99	5.03	40.69	97.70	1.82	52.28	97.75	2.00	70.55	96.23	2.01
S51##	No	Low	90	33	NA	89.17	NA	NA	99.92	NA	NA	97.02	NA	NA	97.06	NA	NA	95.25	NA
S52##	No	Med	90	33	NA	95.65	NA	NA	100.00	NA	NA	96.97	NA	NA	96.98	NA	NA	95.15	NA
S53##	No	Very	38	13	NA	88.28	NA	NA	99.70	NA	NA	97.29	NA	NA	97.03	NA	NA	94.84	NA
S54##	Yes	Low	150	27	NA	98.29	NA	NA	100.00	NA	NA	97.87	NA	NA	97.91	NA	NA	96.07	NA
S55##	Yes	Med	301	79	NA	85.71	NA	NA	99.88	NA	NA	97.65	NA	NA	97.65	NA	NA	95.79	NA
S56##	Yes	Very	3	2	NA	75.16	NA	NA	90.06	NA	NA	96.52	NA	NA	96.52	NA	NA	94.28	NA

*The data set names identify the set of parameters used to generate the simulated data. †A trend over the years was either included or not. ‡Seasonality was included at 3 levels. A dataset that is very seasonal might include pneumonia and influenza. Datasets with less seasonality are medium, and datasets in which we did not intentionally include any seasonality are low. §The means and standard deviations (SD) used in the simulations were based on real means and SDs. ¶The seasonally adjusted CUSUM method sums the positive differences of the current value from the mean for a period similar to the current value over 5 years. #The historical limits method compares the current sum of 4 periods to the mean of the sum of 15, 4 periods surrounding the current point of interest over 5 years. **The C1-MILD method is based on CUSUM, but the calculations reduce to the current value being greater than the mean plus 3 SD, with the mean and standard deviation based on the past 7 days. ††The C2-MEDIUM method is based on CUSUM, but the calculations reduce to the current value being greater than the mean plus 3 SD, with the mean and SD based on the past 7 days shifted by 2 days. ‡‡The C3-ULTRA method is based on CUSUM, summing the positive difference of the current value form the mean for 3 days, with the mean and standard deviation based on the past 7 days shifted by 2 days. §§95% confidence values were <0.06. Time to detection (ttd) must be interpreted with sensitivity because ttd does not include missed outbreaks. ##Datasets S51–S56 do not include any simulated outbreaks; therefore, sen and ttd cannot be calculated.

**Table A2:** Sensitivity (sen) and time to detection (ttd) by method and outbreak type

Type of outbreak	Seasonally adjusted CUSUM*		Historical Limits†		C1–MILD‡		C2–MEDIUM§		C3–ULTRA¶	
	sen (%)	ttd**	sen (%)	ttd**	sen (%)	ttd**	sen (%)	ttd**	sen (%)	ttd**
Spike††	65.55	0.0	10.0	0.0	60.2	0.0	59.9	0.0	63.3	0.0
Spike‡‡	48.3	0.0	7.6	0.0	43.3	0.0	43.1	0.0	47.2	0.0
Inverted log normal§§	92.2	1.3	52.6	2.4	67.2	1.2	82.6	1.4	92.8	1.3
Inverted log normal¶¶	75.9	1.4	30.4	2.3	54.4	1.3	66.0	1.5	80.8	1.5
Inverted log normal##	98.0	2.4	77.2	4.7	66.4	2.5	83.3	2.6	95.1	2.4
Inverted log normal***	88.8	2.8	50.7	4.8	59.4	3.0	71.8	3.1	88.7	3.0
Log normal†††	92.6	0.6	52.5	1.7	70.6	0.4	82.6	0.6	92.5	0.6
Log normal‡‡‡	77.4	0.8	31.4	1.7	54.8	0.6	65.1	0.7	80.1	0.8
Log normal§§§	97.2	1.0	75.8	2.6	67.9	0.8	85.4	1.0	94.3	1.0
Log normal¶¶¶	88.2	1.6	49.9	2.9	56.9	1.2	71.8	1.4	87.2	1.4

*The seasonally adjusted CUSUM method sums the positive differences of the current value from the mean for a period similar to the current value over 5 years. †The historical limits method compares the current sum of 4 periods to the mean of the sum of 15 sets of 4 periods surrounding the current point of interest over 5 years. ‡The C1–MILD method is based on CUSUM, but the calculations reduce to the current value being greater than the mean plus 3 standard deviations (SD), with the mean and SD based on the past 7 days. §The C2–MEDIUM method is based on CUSUM, but the calculations reduce to the current value being greater than the mean plus 3 SD, with the mean and SD based on the past 7 days shifted by 2 days. ¶The C3–ULTRA method is based on CUSUM, summing the positive difference of the current value from the mean for 3 days, with the mean and SD based on the past 7 days shifted by 2 days. **95% confidence values were <0.03. Time to detection must be interpreted with sen because ttd does not include missed outbreaks. ††This type of outbreak represents an increase of 2 SD for 1 day. ‡‡This type of outbreak represents an increase of 3 SD for 1 day. §§This type of outbreak represents an increase of 2 SD with a fast rise in cases with incubation <7days. ¶¶This type of outbreak represents an increase of 3 SD with a fast rise in cases with incubation <7days. ##This type of outbreak represents an increase of 2 SD with a fast rise in cases with incubation >7days. ***This type of outbreak represents an increase of 3 SD with a fast rise in cases with incubation >7days. †††This type of outbreak represents an increase of 2 SD with a slow rise in cases with incubation <7days. ‡‡‡This type of outbreak represents an increase of 3 SD with a slow rise in cases with incubation <7days. §§§This type of outbreak represents an increase of 2 SD with a slow rise in cases with incubation >7days. ¶¶¶This type of outbreak represents an increase of 3 SD with a slow rise in cases with incubation >7days.

**Table:** By method, overall sensitivity and specificity and time to detection

Type of method	Name	Sensitivity (%)	Specificity (%)	Time to detection (d)*
Historical methods (at least 5 y historical data)	Seasonally adjusted CUSUM†	82.5	88.7	1.272
	Historical limits‡	43.9	96.3	2.942
Nonhistorical methods (<3 y historical data)	C1–MILD§	60.1	97.0	1.122
	C2–MEDIUM¶	71.2	97.0	1.319
	C3–ULTRA**	82.3	95.4	1.307

*Time to detection must be interpreted with sensitivity because time to detection does not include missed outbreaks. †The seasonally adjusted CUSUM method sums the positive differences of the current value from the mean for a period similar to the current value over 5 years. ‡The historical limits method compares the current sum of 4 time periods to the mean of the sum of 15 totals of 4 time periods surrounding the current point of interest over 5 years. §The C1–MILD method is based on CUSUM, but the calculations reduce to the current value being greater than the mean plus 3 standard deviations (SD), with the mean and SD based on the past 7 days. ¶The C2–MEDIUM method is based on CUSUM, but the calculations reduce to the current value being greater than the mean plus 3 SD, with the mean and SD based on the past 7 days shifted by 2 days. **The C3–ULTRA method is based on CUSUM, summing the positive difference of the current value from the mean for 3 days, with the mean and SD based on the past 7 days shifted by 2 days.

## References

[1] Teutsch SM, Churchill RE, eds. Principles and practice of public health surveillance. New York: Oxford University Press; 2000.

[2] Stroup DF, Williamson GD, Herndon JL, Karon J. Detection of aberrations in the occurrence of notifiable diseases surveillance data. Stat Med. 1989;8:323–9. 10.1002/sim.4780080312 2540519

[3] Farrington CP, Andrews NJ, Beale AD, Catchpole MA. A statistical algorithm for the early detection of outbreaks of infectious disease. J R Stat Soc Ser A Stat Soc. 1996;159:547–63. 10.2307/2983331

[4] Simonsen L, Clarke JM, Stroup DF, Williamson GD, Arden NH, Cox NJ. A method for timely assessment of influenza-associated mortality in the United States. Epidemiology. 1997;8:390–5. 10.1097/00001648-199707000-00007 9209852

[5] Hutwagner LC, Maloney EK, Bean NH, Slutsker L, Martin SM. Using laboratory-based surveillance data for prevention: an algorithm for detecting salmonella outbreaks. Emerg Infect Dis. 1997;3:395–400. 10.3201/eid0303.970322 9284390 PMC2627626

[6] Stern L, Lightfoot D. Automated outbreak detection: a quantitative retrospective analysis. Epidemiol Infect. 1999;122:103–10. 10.1017/S0950268898001939 10098792 PMC2809594

[7] Hutwagner L, Thompson W, Seeman GM, Treadwell T. The bioterrorism preparedness and response Early Aberration Reporting System (EARS). J Urban Health. 2003;80:i89–96. 12791783 10.1007/PL00022319 PMC3456557

[8] Hutwagner L, Thompson W, Groseclose S, Williamson GD. An evaluation of alternative methods for detecting aberrations in public health surveillance data. American Statistical Association, Joint Statistical Meetings, Proceedings of the Biometrics Section. Indianapolis; 2000 Aug. p. 82–5.

